# Fusariumic Acids I and J, Two New Phytotoxic Isocassadiene-Type Diterpenoids from Tomato Fusarium Crown and Root Rot Pathogen *Fusarium oxysporum* f. sp. *radicis-lycopersici*

**DOI:** 10.3390/toxins18040173

**Published:** 2026-04-03

**Authors:** Prosper Amuzu, Gan Gu, Xuwen Hou, Jiahang Sun, Muhammad Abubakar Jakada, Eromosele Odigie, Daowan Lai, Ligang Zhou

**Affiliations:** State Key Laboratory of Agricultural and Forestry Biosecurity, Department of Plant Pathology, College of Plant Protection, China Agricultural University, Beijing 100193, China; amuzuprosper07@gmail.com (P.A.); gugan@caas.cn (G.G.); xwhou@cau.edu.cn (X.H.); jiahangsun@cau.edu.cn (J.S.); jakada@cau.edu.cn (M.A.J.); eromoseleodigie@cau.edu.cn (E.O.)

**Keywords:** tomato Fusarium crown and root rot, *Fusarium oxysporum* f. sp. *radicis-lycopersici*, isocassadiene-type diterpenoids, fusariumic acids I and J, phytotoxic activity, phytotoxins

## Abstract

*Fusarium oxysporum* f. sp. *radicis-lycopersici* (*Forl*) is the etiological agent of tomato Fusarium crown and root rot (FCRR), a devastating soil-borne disease that severely compromises global tomato production. The pathogenicity of *Forl* has been increasingly linked to its capacity to produce phytotoxic isocassadiene-type diterpenoids. In this study, *Forl* was cultured in rice medium to obtain *Forl* cultures, which were used for the separation and identification of secondary metabolites. After removing the known metabolites, two new isocassadiene-type diterpenoid compounds, namely fusariumic acids I (**1**) and J (**2**), were isolated from the ethyl acetate extract. Their structures were identified using spectroscopic data analyses and quantum chemical calculations. This is the first report of the fusariumic acid analogs containing a hydroxyl group at position C–1 in the molecule. Fusariumic acids I (**1**) and J (**2**) exhibited significantly inhibitory activities on the hypocotyl elongation of tomato (*Solanum lycopersicum*) and sesame (*Sesamum indicum*) seedlings, as well as on the coleoptile elongation of rice (*Oryza sativa* var. *japonica*) seedlings at concentrations from 10 to 100 µg/mL. The discovery of two new phytotoxic isocassadiene-type diterpenoids expanded the diversity of secondary metabolites of *Forl*. Meanwhile, it provided critical insights into *Forl*-tomato interactions and the candidate lead compounds for the development of new herbicides as well.

## 1. Introduction

Tomato (*Solanum lycopersicum*) stands as one of the most economically significant and widely cultivated vegetable crops globally [1,2]. However, tomato production is frequently threatened by more than 200 diseases caused by various pathogenic fungi, bacteria, viruses, and nematodes [3]. Among them, Fusarium crown and root rot (FCRR) caused by the soil-borne fungus *Fusarium oxysporum* f. sp. *radicis-lycopersici* (*Forl*), is particularly destructive in tomato production [4,5]. FCRR has emerged as a major constraint in both greenhouse and field tomato cultivation systems worldwide [6,7,8]. *Forl* infects the host tomato through the roots, causing characteristic symptoms such as brown, necrotic lesions on the taproot and crown, vascular discoloration, wilting, and eventual plant death [9,10]. The persistence of *Forl* in soil and its ability to spread via conidia further complicate disease management [11].

The pathogenicity of *F. oxysporum* is a complex process involving a combination of physical invasion and chemical warfare [5,12]. While some formae speciales, like *F. oxysporum* f. sp. *lycopersici*, rely heavily on secreted effector proteins to suppress host immunity, *Forl* appears to utilize phytotoxic secondary metabolites (known as phytotoxins) as the primary virulence factors [13]. These small molecules can disrupt host cell integrity, interfere with the physiological processes, and facilitate fungal colonization. The chemical arsenal of *Forl* includes a variety of compounds, but the recent attention has focused on a class of isocassadiene-type diterpenoids with phytotoxic activities [13,14]. The first phytotoxic isocassadiene-type diterpenoid named FCRR-Toxin from *Forl* was isolated and identified in 1994 [14]. Recent studies have identified eight such compounds termed fusariumic acids A–H from *Forl* cultures and demonstrated their potent phytotoxicity on tomato seedlings, causing necrosis, and inhibiting root and hypocotyl elongation at low concentrations [13]. As a continuation of our curiosity in the exploration of new phytotoxic secondary metabolites from *Forl*, we carefully investigated the high-performance liquid chromatography, diode array detector, and high-resolution electrospray ionization mass spectrometry (HPLC–DAD–HRESIMS) profile of the ethyl acetate (EtOAc) crude extract of *Forl* fermentation cultures, and found several unidentified peaks that might correspond to new isocassadiene-type diterpenoids.

**Figure 1:**
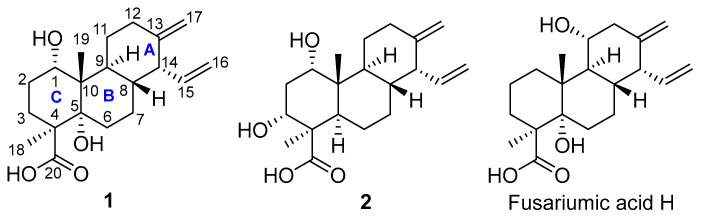
The structures of fusariumic acids I (**1**) and J (**2**).

In this study, *Forl* was cultured in the solid rice medium for one month. Two new phytotoxic diterpenoids, namely fusariumic acids I (**1**) and J (**2**) (Figure 1), were isolated and identified from the extract of *Forl* fermentation cultures. Herein, we report the isolation, structural elucidation, and phytotoxic and cytotoxic activities of these two compounds.

## 2. Results

### 2.1. Structural Identification of Compounds ***1*** and ***2***

The EtOAc crude extract of *Forl* fermentation cultures was subjected to repeated column chromatography over the normal-phase silica gel, reversed-phase silica gel (i.e., ODS), Sephadex LH-20, as well as the semi-preparative HPLC to afford compounds **1** and **2** (Figure 1). The HRESIMS spectra, UV spectra, and 1D and 2D NMR spectra of **1** and **2** are shown in Figures S1–S16. Both compounds were named fusariumic acids I (**1**) and J (**2**), and belonged to isocassadiene-type diterpenoids [15].

#### 2.1.1. Identification of Compound **1**

**Figure 2:**
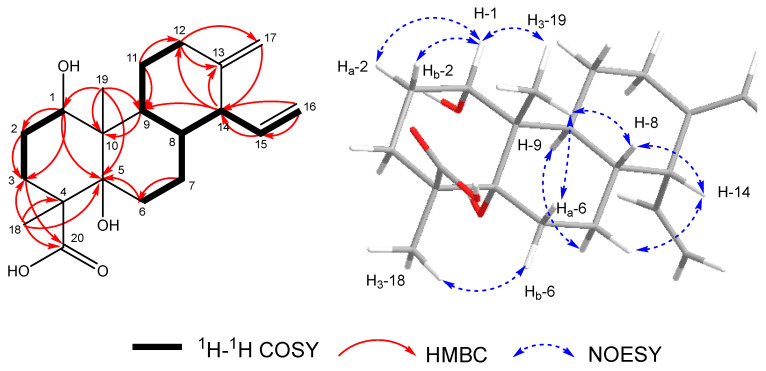
The key ^1^H–^1^H COSY, selected HMBC (H → C), and NOESY correlations of compound **1**.

**Figure 3:**
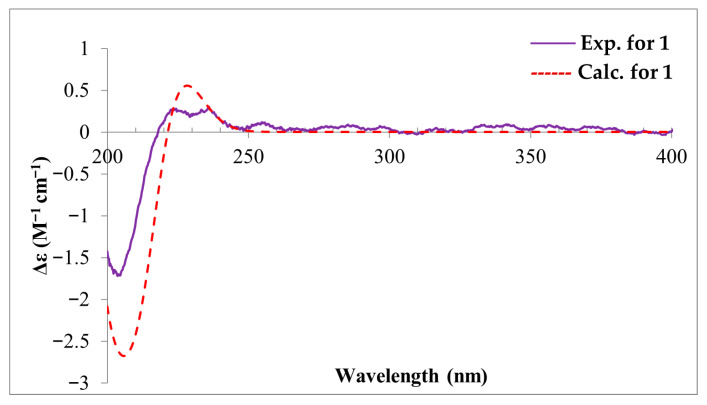
The calculated and experimental ECD spectra of compound **1**.

Compound **1** was isolated as a white amorphous solid. Its molecular formula was designated as C_20_H_30_O_4_ by the HRESIMS spectrum of **1** (Figure S1), which showed a deprotonated molecular ion peak at *m*/*z* 333.2346 [M − H]^−^, indicating six degrees of unsaturation. The characteristics of its UV and MS spectra indicated that it was a diterpenoid by analogy to the co-isolated fusariumic acid H [13] (Figure 1). The ^1^H and ^13^C NMR data of compounds **1** is shown in Table 1, The key ^1^H–^1^H COSY, selected HMBC, and NOE correlations of compound **1** is shown in Figure 2. The calculated and experimental ECD spectra of compound **1** is shown in Figure 3.

Analysis of the ^1^H NMR spectrum (Figure S3) in combination with the HSQC spectrum (Figure S5) of **1** established the presence of several characteristic signals (Table 1). These include two pairs of olefinic methylenes at *δ*_H_ 5.03 (m, H_a_–16)/5.00 (m, H_b_–16), and 4.63 (t, *J* = 2.4 Hz, H_a_–17)/4.54 (t, *J* = 2.4 Hz, H_b_–17), along with an olefinic proton at 6.16 (ddd, *J* = 16.8, 10.4, 9.0 Hz, H–15). A signal for an oxygenated methine proton was observed at 3.77 (t, H–1). Two methyl singlets were present at *δ*_H_ 1.18 (H_3_–18) and 0.78 (H_3_–19). Resonances corresponding to six non-oxygenated aliphatic methylenes and three non-oxygenated aliphatic methines were also discernible. The ^13^C NMR spectrum (Table 1 and Figure S4) exhibited a total of 20 carbon signals. These comprised a carbonyl carbon at *δ*_C_ 181.3, two pairs of olefinic carbons at *δ*_C_ 153.8, 139.1, 116.1, and 106.8, one oxymethine at *δ*_C_ 73.4, one oxygenated sp^3^-hybridized quaternary carbon at *δ*_C_ 80.4, and the remaining resonances were attributed to 13 sp^3^ carbons, including two methyl groups at *δ*_C_ 24.6, 15.2. The carbonyl function and the two double bonds accounted for three degrees of unsaturation. This established a tricyclic structure for compound **1** to fulfill the remaining degrees of unsaturation.

The planar structure was established through 2D NMR (Figures S6 and S7) analysis. Examination of the ^1^H–^1^H COSY correlations of H_2_–12/H_2_–11/H–9/H–8/H–7, combined with key HMBC correlations from H–14 to C–9 and C–12, from H_2_–17 to C–12, C–13, C–14, as well as from H_2_–12 to C–17, delineated the six-membered ring A (Figure 2), confirming that the exocyclic double bond attached to C–13. The vinyl group at C–14 was confirmed by HMBC correlations from H_2_–16 to C–14 and the key ^1^H–^1^H COSY correlations of H_2_–16/H–15/H–14. The six-membered ring B, fused to ring A through C–8 and C–9, was established based on the ^1^H–^1^H COSY correlations (H_2_–6/H_2_–7/H–8/H–9) and key HMBC correlations from H_3_–19 to C–5, C–9, and C–10, from H_2_–6 to C–5, and from H–9 to C–10. The final six-membered ring C, fused to C–5 and C–10, was constructed from the ^1^H–^1^H COSY correlations (H–1/H_2_–2/H_2_–3) and critical HMBC correlations: from H_3_–18 to C–3, C–4, C–5, and C–20; from H_3_–19 to C–1; and from H_2_–3 to C–20. This ring bears a methyl group attached at C–4 and a methyl group attached at C–10, along with a carboxyl group attached at C–4. Finally, the molecular weight of the compound and the carbon chemical shifts of C–1 (*δ*_C_ 73.4) and C–5 (*δ*_C_ 80.4) indicated the presence of hydroxyl groups attached at C–1 and C–5, respectively. Consequently, the planar structure of compound **1** was established.

The relative configuration of **1** was established from analysis of the NOESY spectrum (Figure 2 and Figure S8) and coupling constants within the six-membered rings. The small coupling constant (*J* = 3.0 Hz) for H–1, coupled with an NOESY correlation observed between H–1 and H_2_–2, established H–1 in an equatorial position (assigned as *β*-oriented). The NOESY correlation between H–1 and H_3_–19 indicated that H_3_–19 is also *β*-oriented. Furthermore, NOESY correlations linking H_3_–19/H–8, H–8/H–14, H_3_–19/H_a_–6 (*δ*_H_ 2.19), and H–14/H_b_–7 (*δ*_H_ 1.21), demonstrated their shared *β*-orientation, revealing the α-orientation of the vinyl group at C–14. While the NOESY correlations between H_3_-18/H_b_–6 (*δ*_H_ 1.74), and H–9/H_a_–7 (*δ*_H_ 1.78) allowed the assignment of H–9 and H_3_–18 as *α*-oriented. Considering the well-documented configurations of isocassadiene-type diterpenoids reported in the literature [13], combined with biosynthetic pathway reasoning, the hydroxyl group at C–5 was deduced to be *α*-oriented.

The absolute configuration of **1** was determined by ECD calculation at PBE0/TZVP//B3PW91/6-311g(d) level, using the solvent model (PCM = MeOH). The calculated ECD spectrum of **1** matched the experimental ECD well (Figure 3). Therefore, the absolute configuration of **1** was assigned as 1*S*, 4*S*, 5*S*, 8*R*, 9*S*, 10*S*, 14*R*, and compound **1** was designated as fusariumic acid I.

#### 2.1.2. Identification of Compound **2**

**Figure 4:**
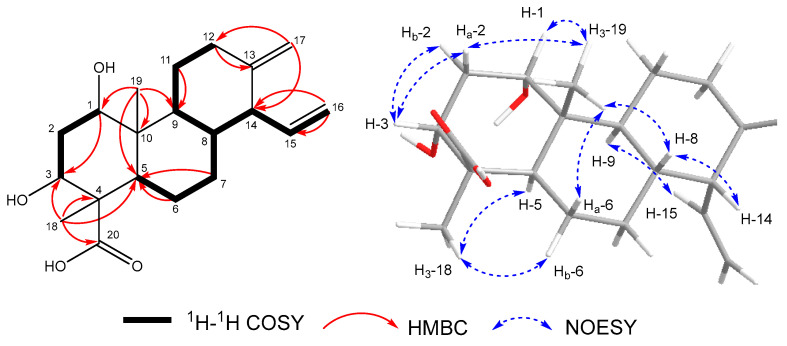
The key ^1^H–^1^H COSY, selected HMBC (H → C), and NOESY correlations of compound **2**.

**Figure 5:**
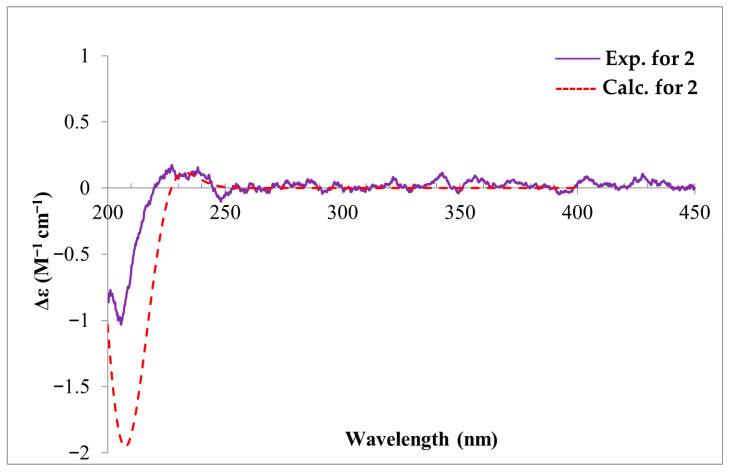
The calculated and experimental ECD spectra of compound **2**.

The ^1^H and ^13^C NMR data of compounds **2** is shown in Table 2, The key ^1^H–^1^H COSY, selected HMBC, and NOE correlations of compound **2** is shown in Figure 4. The calculated and experimental ECD spectra of compound **2** is shown in Figure 5. The HRESIMS data established that compound **2** shared the same molecular formula (C_20_H_30_O_4_) as compound **1**. The detailed analysis of its 1D NMR data (Table 2) revealed that it also exhibited the characteristic skeleton of an isocassadiene-type diterpenoid, closely resembling compound **1**. The primary structural difference involved the absence of the hydroxyl group at C–5 and the presence of a new hydroxyl group substituent at C–3. The loss of the C–5 hydroxyl group was deduced from the following key observations: the ^1^H–^1^H COSY correlations (H–5/H_2_–6/H_2_–7), the HMBC correlation from H_2_–7 to C–5, and the HMBC correlation from H_3_–18 to C–5. The hydroxyl substitution at C–3 was confirmed by the ^1^H–^1^H COSY correlations (H–1/H_2_–2/H–3) in conjunction with the downfield chemical shift observed for C–3 (*δ*_C_ 72.9). Compound **2** displayed NOESY correlations analogous to those observed in compound **1** for key chiral centers (C–1, C–4, C–8, C–9, C–10, C–14), indicating the same relative configuration within the conserved core (Figure 4 and Figure S16). For the stereochemistry at C–3, the small coupling constant (*J* = 3.2 Hz) for H–3, coupled with an observed NOESY correlation between H–3 and H_2_–2, established H–3 in an equatorial position (*β* orientation). Hence, as observed in all the reported isocassadienes, a *trans*-coupling occurred between rings B and C. Furthermore, an NOESY correlation between H–5 and H_3_–28 indicated that these protons share the same orientation (*α* orientation). The calculated ECD spectrum of **2** matched the experimental ECD well (Figure 5). Therefore, the absolute configuration of **2** was assigned as 1*S*, 3*R*, 4*R*, 5*R*, 8*R*, 9*S*, 10*R*, 14*R*, and compounds **2** was designated as fusariumic acid J.

### 2.2. Phytotoxic Activity of Compounds ***1*** and ***2***

**Figure 6:**
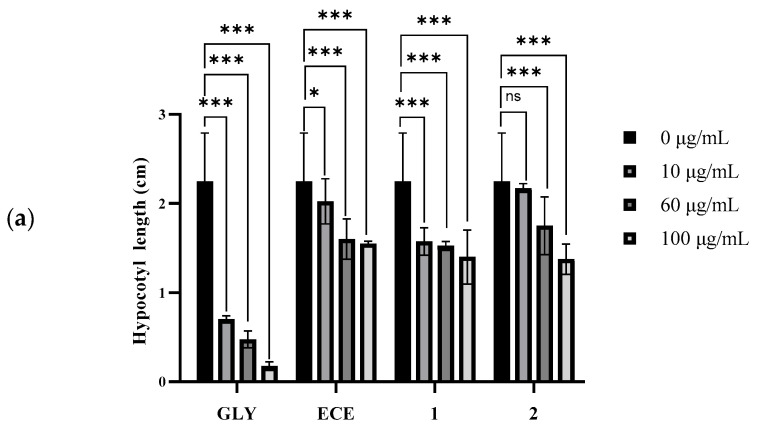

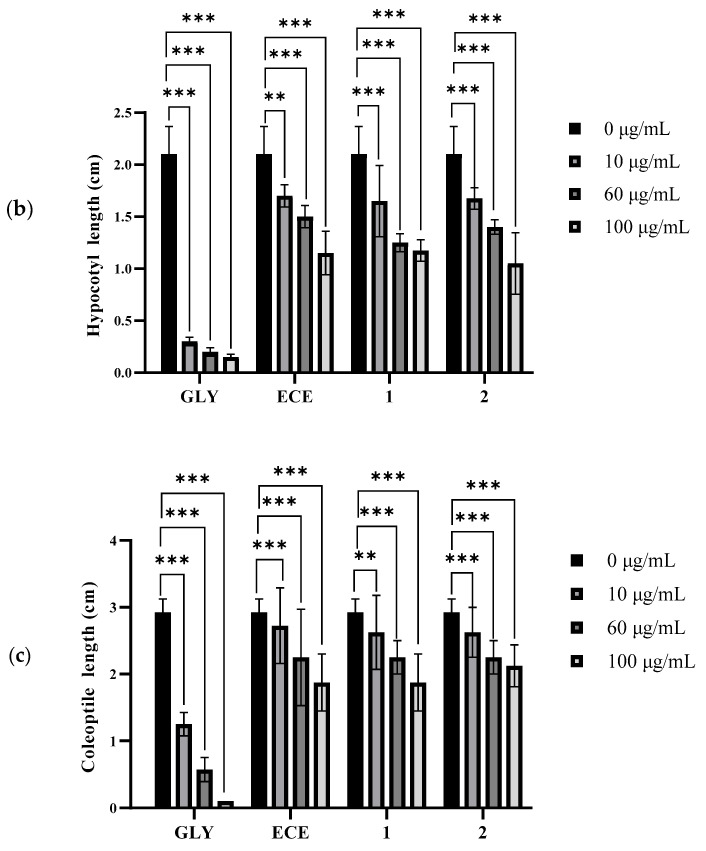
The effects of compounds **1** and **2**, EtOAc crude extract (ECE), and the positive control glyphosate (GLY) on the hypocotyl elongation of tomato (**a**) and sesame (**b**) seedlings, and coleoptile elongation of rice seedlings (**c**), respectively. Each datum was the average of three replicates ± standard deviation (SD). The statistically significant differences at *p* > 0.05 (ns), *p* ≤ 0.05 (*), *p* ≤ 0.01 (**), and *p* ≤ 0.001 (***) were determined for the radicle length of seedlings treated with 10 μg/mL, 60 μg/mL, and 100 μg/mL of samples compared with the 0 μg/mL.

The EtOAc crude extract (ECE) and compounds **1** and **2** were examined for their phytotoxicities on three plant seedlings and cytotoxicities on six human cancer cell lines. The positive controls for the evaluation of phytotoxic and cytotoxic activities were glyphosate (GLY) and taxol, respectively. The effects of the compounds **1** and **2**, ECE, and positive control GLY on the hypocotyl elongation of dicotyledonous tomato and sesame seedlings, and coleoptile elongation of monocotyledonous rice seedlings, are shown, respectively, in Figure 6a, Figure 6b, and Figure 6c. Correspondingly, the images of the compounds **1** and **2**, ECE and GLY affecting the growth of tomato, sesame, and rice seedlings are shown in Figures S17, S18, and S19, respectively.

Compounds **1** and **2**, and ECE all exhibited significant inhibition activities on the hypocotyl elongation of tomato and sesame seedlings, as well as on the coleoptile elongation of rice seedlings at concentrations from 10 µg/mL to 100 µg/mL. Their inhibitory activities showed obvious dose-effect relations. The inhibitory rates of two compounds (**1** and **2**) and ECE ranged from 6.8% to 50.0%, which were weaker than those of the positive control GLY at the same concentration. When the concentration of compound **2** was at 100 µg/mL, the inhibitory rate reached the maximum value (50.0%) on the hypocotyl elongation of sesame seedlings, as the maximum inhibition reached approximately 50% at 100 μg/mL, which indicated the modest activity of fusariumic acids I (**1**) and J (**2**). The EC_50_ values were not calculated as the inhibition did not consistently reach 50% across conditions, making the curve fitting unreliable. Fusariumic acids I (C–1 hydroxyl) and J (C–1/C–3 hydroxyls) were the first C–1 hydroxylated analogs. Compared to fusariumic acids A–H, the C–1 hydroxylation of fusariumic acids I (**1**) and J (**2**) maintained phytotoxicity. The comprehensive structure-activity relationship (SAR) analysis required more derivatives, which has not been carried out.

The results of this study, combined with the previous findings [13], indicated that these isocassadiene-type diterpenoids should be the main phytotoxic components in the ECE of *Forl* fermentation cultures. However, their phytotoxic mechanisms need further investigation.

Compounds **1** and **2** were also assessed for their cytotoxic activities, with IC_50_ values shown in Table S1. However, neither compound showed any significant cytotoxic activities on six human cancer cell lines, with IC_50_ values all greater than 50 µM, in contrast to the positive control taxol with IC_50_ values from 0.00007 µM to 0.028 µM. This indicated that compounds **1** and **2** exhibited phytotoxicity, but not obvious mammalian cytotoxicity.

## 3. Discussion

### 3.1. Diversity of Forl Secondary Metabolites

Fusariumic acids I (**1**) and J (**2**) shared an identical molecular formula (C_20_H_30_O_4_) and tricyclic isocassane core. Fusariumic acid I (**1**) featured a single hydroxyl group at C–1, while fusariumic acid J (**2**) had an additional hydroxyl group at C–3. To our knowledge, the fusariumic acid analogs containing a hydroxyl group at the position C–1 in the molecule have not been reported previously [13]. The discovery of fusariumic acids I (**1**) and J (**2**) significantly increased the chemical diversity of isocassane-type diterpenoids in the phytopathogenic fungus *Forl*. This subtle structural variation likely arose from the catalysis of cytochrome P450 enzymes that modify a common isocassane precursor [13]. Such metabolic plasticity enabled *Forl* to generate chemical diversity without fundamentally altering its core scaffold, which might enhance the pathogenicity and adaptability of *Forl* during its host colonization and interactions with the environmental factors, such as the rhizosphere microorganisms and various stresses [14]. However, the mechanisms of these diterpenoids in the interactions between *Forl* and tomato plants need further investigation, such as their physiological effects on host plants (i.e., pathogenicity, production of reactive oxygen species, membrane integrity, mitochondrial function, and hormone interference), and the biosynthetic regulation linking the phytotoxin production to FCRR development.

### 3.2. Management of Tomato FCRR and Forl Phytotoxins

The management strategies for FCRR have traditionally relied on soil fumigation, crop rotation, grafting, and the use of resistant cultivars [4,16,17,18,19]. However, the environmental concerns associated with chemical fumigants and the emergence of new pathogen races have spurred research into alternative, sustainable control methods. These include biological control using antagonistic microorganisms like *Trichoderma* spp. [20] and *Bacillus* spp. [21], plant extracts and secondary metabolites [22,23], as well as the application of soil amendments such as compost [24,25] and biochar [26]. Despite these advances, FCRR remains a persistent threat, highlighting the need for a further understanding of the pathogen’s virulence mechanisms to develop more effective control strategies.

*Forl* relies predominantly on the effector proteins to suppress host immunity. It appeared to deploy small-molecule phytotoxins as the key virulence determinants [5]. The persistent identification of phytotoxic diterpenoids from the initially identified FCRR-Toxin [14] to the recently revealed fusariumic acids A–J [13] supported the hypothesis that chemical warfare played a central role in *Forl*’s infection strategy. This insight opened avenues for novel control approaches which target *Forl* diterpenoid phytotoxin biosynthesis. Understanding the biosynthetic pathway and identifying the key enzymes involved, it will provide new targets (i.e., the casbene synthase and P450 monooxygenases) for blocking the biosynthesis of phytotoxins in *Forl*. This will provide a basis for the prevention and control of *Forl* diterpenoid phytotoxins and tomato FCRR [27].

### 3.3. Biological Activities and Application Potentials of Forl Diterpenoids

*Forl* was known to produce fusaric acid, a host non-specific phytotoxin that disrupted the cell membrane integrity and mitochondrial functions of tomato and other plant species, contributing to wilting and necrosis [28]. This study revealed two additional new isocassane-type diterpenoids, fusariumic acids I (**1**) and J (**2**) from *Forl*. Both compounds showed the obvious inhibitory activities on the hypocotyl elongation of host tomato seedlings, which was consistent with the previous results [13]. In addition, they showed phytotoxic activities on other plants such as the dicotyledonous sesame and monocotyledonous rice plants, which indicated that isocassane-type diterpenoids also belonged to the host non-specific phytotoxins [29,30]. Their phytotoxic activities on plants and non-cytotoxic activities on mammalian cells indicated a plant-specific mode of action that distinguishes these diterpenoids from general biocides [31]. This will provide candidate lead compounds for the creation of broad-spectrum herbicides for weed control [32,33].

## 4. Conclusions

This study revealed two new isocassadiene-type diterpenoids, fusariumic acids I (**1**) and J (**2**) from *Forl*. Their planar structures and absolute configurations were rigorously determined through integrated spectroscopic and TDDFT-calculated ECD analysis. Biological activity assessment showed that both compounds exhibited obvious phytotoxicity against tomato, sesame, and rice seedlings. This is the first report of the fusariumic acid analogs containing a hydroxyl group at the position C–1 in the molecule. The findings expand the structural diversity of this emerging class of fungal phytotoxins. The subsequent investigation includes the biosynthesis and phytotoxic action mechanisms of fusariumic acid analogs. The identified key enzymes in the biosynthesis of these diterpenoids will provide new targets for the creation of fungicides to block the production of phytotoxins in *Forl* [34,35]. In addition, fusariumic acids I (**1**) and J (**2**) are host non-specific phytotoxins that exhibit no cytotoxicity on mammalian cells. This will highlight the potential of these structurally unique diterpenoids as leads for the development of eco-friendly herbicides [36,37].

## 5. Materials and Methods

### 5.1. Fungal Strain and Culture Conditions

The Foxy-SG strain of FCRR pathogen was obtained from Prof. Lihua Geng at the Vegetable Research Center of the Beijing Academy of Agriculture and Forestry Sciences, China. The fungus was verified as *F. oxysporum* f. sp. *radicis-lycopersici* (*Forl*) based on the ITS-rDNA sequencing (GenBank accession number: HQ603748) [38], and microscopic examination that was the same as described in the literature [39]. For preservation, the strain was kept at −80 °C. To activate the fungus, the mycelia were cultured on potato dextrose agar (PDA) and maintained in darkness at 28 °C for one week. A seed culture was subsequently produced by transferring roughly 5 mL of mycelia suspension into a 250 mL Erlenmeyer flask containing 100 mL of potato sucrose broth (PSB). The flasks were shaken at 180 rpm and 28 °C for 7 days. For batch fermentation, 3.0 kg of rice grains was distributed across thirty 1000 mL flasks. Each flask was filled with 100 g of rice grains and 110 mL of distilled water, allowed to soak overnight, and sterilized. The solid rice medium in each flask was inoculated with the seed cultures at 28 °C in the dark for 30 days.

### 5.2. Extraction and Isolation

In order to isolate compounds, the harvested fermentation cultures were fragmented, air-dried, and pulverized into a fine powder. The powder was extracted exhaustively with ethyl acetate (EtOAc) at ambient temperature. The resulting leachate was passed through filter paper and concentrated via a rotary evaporation under reduced pressure, yielding 247 g of EtOAc crude extract (ECE).

The general separation and purification of the compounds included column chromatography (CC) and semi-preparative HPLC separation. The CC materials included Sephadex LH-20 (40–70 μm; Amersham Pharmacia Biotech, Uppsala, Sweden), positive phase silica gel (200–300 mesh, Qingdao Marine Chemical Inc., Qingdao, China), and reversed-phase (RP)-C_18_ silica gel (20–45 μm, Fuji Silysia Chemical Ltd., Aichi, Japan). The analytical HPLC-DAD was conducted on a Shimadzu LC-20A equipped with an SPD-M20A photodiode array detector (Shimadzu Corp., Tokyo, Japan) and a Phenomenex analytic C_18_ column (250 mm × 4.6 mm i.d., 5 μm; Torrance, CA, USA). The semi-preparative HPLC separation was carried out on a Lumtech system (Lumiere Tech. Ltd., Beijing, China) featuring a K-501 pump (flow rate, 3 mL/min) and a K-2501 UV detector using a Luna-C_18_ column (250 mm × 10 mm i.d., 5 μm, Phenomenex Inc., Torrance, CA, USA). In this study, HPLC-DAD was employed primarily for the qualitative analysis of compound purity and profiling. Although quantitative analysis was not the primary objective, method validation was performed to establish sensitivity limits and extraction recovery. This ensures the reproducibility of the chromatographic analysis and confirms the integrity of the isolated compounds used for bioassays. The limit of detection (LOD) and limit of quantification (LOQ) were determined based on the signal-to-noise ratios (S/N) of 3:1 and 10:1, respectively. The LOD was established at 0.1 µg/mL, and the LOQ at 0.5 µg/mL. Accuracy was assessed via spike-recovery experiments with a recovery rate ranging from 92% to 98% for compounds **1** and **2**.

The ECE was fractionated through the vacuum liquid chromatography (VLC) on a positive phase silica gel column (10 cm × 100 cm). The elution was carried out by a stepwise gradient of petroleum ether (PE) and EtOAc, followed by EtOAc and methanol (CH_3_OH), with increasing polarity [PE/EtOAc (1:0, 50:1, 30:1, 10:1, 10:2, 3:1, 10:5, 1:1, 1:2, 0:1, *v*/*v*) and EtOAc/CH_3_OH (5:1, 3:1, 2:1, 1:1, *v*/*v*)]. Fractions were collected and analyzed by analytical HPLC. Based on their HPLC profiles, fractions were combined into 13 major fractions (i.e., Frs. 1–13).

Fractions 5–13, which showed complex chemical profiles, were combined (33.73 g) and subjected to Sephadex LH-20 chromatography using a CH_2_Cl_2_/CH_3_OH (1:1, *v*/*v*) solvent mixture, yielding 13 subfractions, which were further subjected to reverse-phase ODS column separation with a stepwise gradient of methanol in water (from 25% to 80% CH_3_OH). This process yielded 31 subfractions (designated F8.5.1 through F8.5.31).

Subfraction F8.5.18 (128.9 mg) was separated using an isocratic mobile phase of MeCN/H_2_O (50:50, *v*/*v*) at a flow rate of 3 mL/min by semi-preparative HPLC, yielding compound **1** (2.7 mg, Rt = 32.14 min). Subfraction F8.5.20 (332.2 mg) was separated with MeCN/H_2_O (55:45, *v*/*v*) at 3 mL/min, yielding compound **2** (8.2 mg, Rt = 33.62 min). By measuring the HPLC profile, UV absorption, and LC–MS data of each compound, and comparing them with the authentic compounds, the others were identified as the known compounds [13].

### 5.3. General Structural Elucidation

The spectroscopic analyses were employed to elucidate the structures of the purified compounds. The UV adsorption measurements were conducted using a TU−1810 UV−vis spectrophotometer (Beijing Persee General Instrument Co., Ltd., Beijing, China). The high-resolution electrospray ionization mass spectrometry (HRESIMS) data were acquired on an Agilent LC 1260−Q−TOF/MS 6520 system (Agilent Technologies, Santa Clara, CA, USA). ^1^H, ^13^C, and 2D NMR experiments (HSQC, HMBC, ^1^H–^1^H COSY, and NOESY) were performed on a Bruker Avance 500 NMR spectrometer (Bruker BioSpin, Zürich, Switzerland). Chemical shifts were calibrated in *δ* (ppm) relative to the solvent peak signals at *δ*_H_ 3.31, and *δ*_C_ 49.0 for deuterated methanol (CD_3_OD), and *δ*_H_ 2.05, and *δ*_C_ 29.9, and 206.7 for deuterated acetone (CD_3_COCD_3_). The coupling constants (*J*) were in hertz (Hz). The optical rotation values were obtained using a Rudolph Autopol IV automatic polarimeter (Rudolph Research Analytical, Hackettstown, NJ, USA).

Fusariumic acid I (**1**): white, amorphous solid; [α]^25^_D_ + 26.9 (c 0.3, MeOH); ECD (*c* = 8.19 × 10^−4^ M, MeOH) λ 206.9 nm; UV (MeOH) λ_max_ 226 nm; ^1^H NMR (CD_3_OD, 500 MHz) see Table 1, _13_C NMR (CD_3_OD, 125 MHz) see Table 1; HRESIMS *m*/*z* 333.2346 [M − H]^−^.

Fusariumic acid J (**2**): white, amorphous solid; [α]^25^_D_ + 50.0 (c 0.06, MeOH); ECD (c = 8.67 × 10^−4^ M, MeOH) λ 200.9, 222.4 nm; UV (MeOH) λ_max_ 228 nm; ^1^H NMR (CD_3_OD, 500 MHz) see Table 1, _13_C NMR (CD_3_COCD_3_), 125 MHz) see Table 1; HRESIMS *m*/*z* 333.2265 [M − H]^−^.

### 5.4. Calculation of ECD

Electronic circular dichroism (ECD) spectra were acquired using a JASCO J−1500 CD spectrometer (JASCO Corp., Tokyo, Japan). The absolute configurations of fusariumic acid I (**1**) and J (**2**) were established by correlating experimental ECD spectra with those calculated using quantum chemically calculated spectra according to the previous report [40]. Briefly, the conformers of compounds **1** and **2** were initiated using Spartan 14 (v1.1.4) employing the MMFF94 force field with an energy cutoff of 3.0 kcal/mol [41]. The subsequent DFT computations were carried out using Gaussian 16 (E.01). The geometry optimization and frequency analyses were conducted at the B3PW91/6-311g(d) level. Theoretical ECD (TDDFT) of each isolated compound was calculated at the PBE0/TZVP level with the IEF−PCM solvent (MeOH) model as well. The special simulations were generated using SpecDis v1.70.1 with a σ/γ value of 0.3 eV [42]. The final spectra were derived by Boltzmann-averaging of individual conformer curves according to their Gibbs free energies. The calculated ECD spectra were UV−shifted by +10 nm for comparison with the measured spectrum.

### 5.5. Phytotoxicity Assay

The phytotoxic activities of the crude extract and isolated compounds were assessed using a seedling elongation bioassay on tomato (*Solanum lycopersicum* cv. Jingkang 4001), sesame (*Sesamum indicum*), and rice (*Oryza sativa* var. *japonica* cv. Daohuaxiang) according to the previous method [13,43]. Briefly, the surface-sterilization was carried out on the seeds with 1% sodium hypochlorite, rinsed with sterilized water, and placed on filter paper in 24-well plates (3 seeds per well). The test compounds **1** and **2** were dissolved in methanol and diluted with sterile water to final concentrations ranging from 10 to 100 µg/mL (final methanol concentration ≤ 1%). Control wells received the solvent mixture only. The plates were sealed and incubated in a growth chamber at 28 ± 1 °C with a 16 h/8 h light/dark cycle for a period of 120 h for tomato seedlings, 144 h for sesame seedlings, and 168 h for rice seedlings, respectively. After incubation, the hypocotyl elongation of tomato and sesame seedlings, and coleoptile elongation of rice seedlings were measured for their lengths, as the hypocotyl/coleoptile elongation was the most sensitive at compound concentrations ranging from 10 to 100 µg/mL. Glyphosate was used as the positive control. The inhibition rate was calculated as [1 − (mean length of treatment/mean length of control)] × 100. All experiments were performed in triplicate.

### 5.6. Cytotoxicity Assay

The cytotoxicity assay mainly referred to the previous methods [44]. The cytotoxic activities of the purified compounds were estimated against HCT116 colon carcinoma cell line (ATCC number: CL1455), U87 MG glioblastoma cell line (ATCC number: CL1485), BGC823 gastric carcinoma cell line (ATCC number: TCP-1008), HepG2 hepatocellular carcinoma cell line (ATCC number: CL1353), PC-9 lung adenocarcinoma cell line (ATCC number: CRL-2868), and PANC1 pancreatic carcinoma cell line (ATCC number: CRL-1469) as a panel of human cancer cell lines. They were bought from the American Type Culture Collection (ATCC) (accessed on 1 April 2025 at https://www.atcc.org). The cells were cultured in appropriate media (DMEM or RPMI-1640) supplemented with 10% fetal bovine serum (FBS) and 1% penicillin-streptomycin at 37 °C in a humidified atmosphere with 5% CO_2_. The cytotoxicity was assessed using the MTT (3-(4,5-dimethylthiazol-2-yl)-2,5-diphenyltetrazolium bromide) assay. The cells were plated in 96-well plates at a density of 5000 cells/well and incubated overnight to allow for attachment. The cells were subsequently treated with serial dilutions of the test compounds (final concentrations ranging from 0.1 to 100 µM) for 48 h. After incubation, MTT solution was added, and the resulting formazan crystals were solubilized in DMSO. Absorbance was then measured at 570 nm. The median inhibitory concentration (IC_50_, μmol/L) of samples on cell growth was calculated based on the linear relation between the inhibitory probability and the logarithm of concentration. Taxol was included as a positive control.

### 5.7. Statistical Analysis

All experiments were planned using three independent biological replicates, with technical triplicates performed for each treatment. The plant seedlings exposed to the test compounds were analyzed at consistent incubation time points. Individual samples exhibiting hypocotyl/coleoptile lengths < 2 mm at the time of measurement or visible mechanical damage unrelated to treatment were excluded. The statistical analyses and diagrams were performed using GraphPad Prism (version 8.0.2) software manufactured by GraphPad Software Inc. (San Diego, CA, USA). The data from the phytotoxicity assay were presented as the mean ± standard deviation (SD) of three independent replicates. The significant differences were assessed by one-way ANOVA followed by Tukey’s multiple comparisons test with significant thresholds defined as *p* > 0.05 (ns), *p* ≤ 0.05 (*), *p* ≤ 0.01 (**), and *p* ≤ 0.001 (***) in the same treatment.

## Figures and Tables

**Table 1:** The ^1^H NMR (500 MHz) and ^13^C NMR (125 MHz) data of **1** (CD_3_OD).

Position	*δ*_H_, Mult. (*J* in Hz)	*δ*_C_, Type
1	3.77 t (3.0)	73.4 CH
2	2.26 m; 1.61 m	28.1 CH_2_
3	1.80 m; 1.76 m	27.8 CH_2_
4		50.4 C
5		80.4 C
6	2.19 m; 1.74 m	29.4 CH_2_
7	1.78 m; 1.21 m	27.0 CH_2_
8	1.57 m	41.1 CH
9	2.49 td (12.1, 3.5)	36.1 CH
10		44.7 C
11	1.84 m; 1.08 dd (12.6, 4.3)	28.0 CH_2_
12	2.26 m; 2.17 m	32.4 CH_2_
13		153.8 C
14	2.85 dd (9.0, 4.4)	56.3 CH
15	6.16 dd (16.8, 10.4, 9.0)	139.1 CH
16	5.03 m; 5.00 m	116.1 CH_2_
17	4.63 d (2.4); 4.54 d (2.4)	106.8 CH_2_
18	1.18 s	24.6 CH_3_
19	0.78 s	15.2 CH_3_
20		181.3 C

Note: The signals were further confirmed through the HMBC spectrum.

**Table 2:** The ^1^H NMR (500 MHz) and ^13^C NMR (125 MHz) data of **2** (CD_3_COCD_3_).

Position	*δ*_H_, Mult. (*J* in Hz)	*δ*_C_, Type
1	3.72 t (3.2)	73.2 CH
2	2.32 dt (14.9, 3.2); 1.92 m	32.4 CH_2_
3	4.03 t (3.2)	72.9 CH
4		48.6 C
5	1.79 m	43.7 CH
6	1.91 m; 1.84 m	23.7 CH_2_
7	1.50 m; 1.20 ddt (13.1, 12.5, 4.5)	33.4 CH_2_
8	1.53 m	41.7 CH
9	2.02 m	40.7 CH
10		42.5 C
11	1.83 m; 1.01 m	27.4 CH_2_
12	2.18 m; 2.11 m	31.9 CH_2_
13		153.4 C
14	2.86 m	56.0 CH
15	6.10 dt (16.9, 9.7)	138.9 CH
16	5.00 m; 4.98 m	115.9 CH_2_
17	4.62 d (2.0); 4.52 d (2.0)	106.6 CH_2_
18	1.29 s	24.7 CH_3_
19	0.71 s	13.3 CH_3_
20		179.3 C

Note: The signals were further confirmed through the HMBC spectrum.

## Data Availability

The original contributions presented in this study are included in the article/Supplementary Material. Further inquiries can be directed to the corresponding author.

## Supplementary Materials

The following supporting information can be downloaded at: https://www.mdpi.com/article/10.3390/toxins18040173/s1, Figure S1: The HRESIMS spectrum of fusariumic acid I (**1**); Figure S2: The UV spectrum of fusariumic acid I (**1**); Figure S3: The ^1^H NMR spectrum of fusariumic acid I (**1**); Figure S4: The ^13^C NMR spectrum of fusariumic acid I (**1**); Figure S5: The HSQC spectrum of fusariumic acid I (**1**); Figure S6: The ^1^H–^1^H COSY spectrum of fusariumic acid I (**1**); Figure S7: The HMBC spectrum of fusariumic acid I (**1**); Figure S8: The NOESY spectrum of fusariumic acid I (**1**); Figure S9: The HRESIMS spectrum of fusariumic acid J (**2**); Figure S10: The UV spectrum of fusariumic acid J (**2**); Figure S11: The ^1^H NMR spectrum of fusariumic acid J (**2**); Figure S12: The ^13^C NMR spectrum of fusariumic acid J (**2**); Figure S13: The HSQC spectrum of fusariumic acid J (**2**); Figure S14: The ^1^H–^1^H COSY spectrum of fusariumic acid J (**2**); Figure S15: The HMBC spectrum of fusariumic acid J (**2**); Figure S16: The NOESY spectrum of fusariumic acid J (**2**); Figure S17: The effects of fusariumic acids I (**1**) and J (**2**), EtOAc crude extract and glyphosate on the growth of tomato seedlings for a period of 5 days; Figure S18: The effects of fusariumic acids I (**1**) and J (**2**), EtOAc crude extract and glyphosate on the growth of sesame seedlings for a period of 6 days; Figure S19: The effects of fusariumic acids I (**1**) and J (**2**), EtOAc crude extract and glyphosate on the growth of rice seedlings for a period of 7 days; Table S1: The cytotoxic activities of fusariumic acids I (**1**) and J (**2**) on human cancer cell lines.

## Abbreviations

The following abbreviations are used in this manuscript:

ATCC	American Type Culture Collection
^13^C NMR	carbon nuclear magnetic resonance spectrum
CC	column chromatography
CD_3_OD	deuterated methanol
CD_3_COCD_3_	deuterated acetone
DAD	diode array detector
ECE	ethyl acetate crude extract
ECD	electronic circular dichroism
EtOAc	ethyl acetate
FCRR	Fusarium crown and root rot
*Forl*	*Fusarium oxysporum* f. sp. *radicis-lycopersici*
GLY	glyphosate
^1^H NMR	proton nuclear magnetic resonance spectrum
^1^H–^1^H COSY	homonuclear chemical shift correlated spectroscopy
HMBC	heteronuclear multiple bond coherence spectrum
HPLC	high-performance liquid chromatography
HSQC	heteronuclear singular quantum correlation spectrum
HRESIMS	high-resolution electrospray ionization mass spectrometry
IC_50_	median inhibitory concentration
*J*	coupling constant
MTT	3-(4,5-dimethylthiazol-2-yl)-2,5-diphenyltetrazolium bromide
NOE	nuclear Overhauser effect
NOESY	nuclear Overhauser effect spectroscopy
PDA	potato dextrose agar
PSB	potato sucrose broth
Rt	retention time
SAR	structure-activity relationship
TDDFT	time-dependent density functional theory
VLC	vacuum liquid chromatography
