# Supplementary material for: Fusariumic Acids I and J, Two New Phytotoxic Isocassadiene-Type Diterpenoids from Tomato Fusarium Crown and Root Rot Pathogen Fusarium oxysporum f. sp. radicis-lycopersici
Source: Toxins (Basel). 2026 Apr 3;18(4):173. doi: 10.3390/toxins18040173 (PMC13120249; doi:10.3390/toxins18040173)
Supplement: Supplementary file 1 [file toxins-18-00173-s001.zip › toxins-4171444-supplementary.pdf]

Supplementary Materials: Fusariumic Acids I and J, Two New Phytotoxic Isocassadiene-Type Diterpenoids from Tomato Fusarium Crown and Root Rot Pathogen *Fusarium oxysporum* f. sp. *radicis-lycopersici*

Prosper Amuzu, Gan Gu, Xuwen Hou, Jiahang Sun, Muhammad Abubakar Jakada, Eromosele Odigie, Daowan Lai and Ligang Zhou

Contents

1. The HRESIMS, UV, 1D and 2D NMR spectra of fusariumic acid I (1) 2

Figure S1. The HRESIMS spectrum of fusariumic acid I (**1**). 2

Figure S2. The UV spectrum of fusariumic acid I (**1**). 2

Figure S3. The ^{1}H NMR spectrum of fusariumic acid I (**1**). 3

Figure S4. The ^{13}C NMR spectrum of fusariumic acid I (**1**). 4

Figure S5. The HSQC spectrum of fusariumic acid I (**1**). 5

Figure S6. The ^{1}H–^{1}H COSY spectrum of fusariumic acid I (**1**). 6

Figure S7. The HMBC spectrum of fusariumic acid I (**1**). 7

Figure S8. The NOESY spectrum of fusariumic acid I (**1**). 8

2. The HRESIMS, UV, 1D and 2D NMR spectra of fusariumic acid J (2) 9

Figure S9. The HRESIMS spectrum of fusariumic acid J (**2**). 9

Figure S10. The UV spectrum of fusariumic acid J (**2**). 9

Figure S11. The ^{1}H NMR spectrum of fusariumic acid J (**2**). 10

Figure S12. The ^{13}C NMR spectrum of fusariumic acid J (**2**). 11

Figure S13. The HSQC spectrum of fusariumic acid J (**2**). 12

Figure S14. ^{1}H–^{1}H COSY spectrum of fusariumic acid J (**2**). 13

Figure S15. The HMBC spectrum of fusariumic acid J (**2**). 14

Figure S16. The NOESY spectrum of fusariumic acid J (**2**). 15

3. The phytotoxic and cytotoxic activities of fusariumic acids I (1) and J (2) 16

Figure S17. The effects of fusariumic acids I (**1**) and J (**2**), EtOAc crude extract (ECE) and glyphosate (GLY) on the growth of tomato seedlings for a period of 5 days. 16

Figure S18. The effects of fusariumic acids I (**1**) and J (**2**), EtOAc crude extract (ECE) and glyphosate (GLY) on the growth of sesame seedlings for a period of 6 days. 17

Figure S19. The effects of fusariumic acids I (**1**) and J (**2**), EtOAc crude extract (ECE) and glyphosate (GLY) on the growth of rice seedlings for 7 days. 18

Table S1. The cytotoxic activities of fusariumic acids I (**1**) and J (**2**) on human cancer cell lines. 19

1. The HRESIMS, UV, 1D and 2D NMR spectra of fusariumic acid I (1)

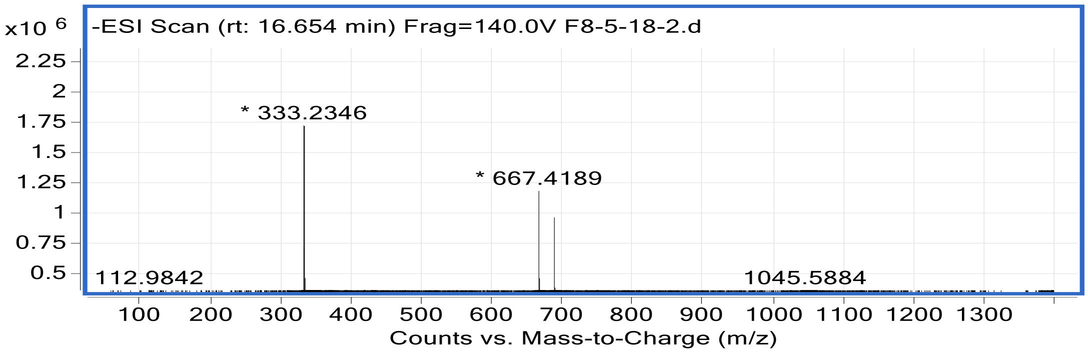

Figure S1. The HRESIMS spectrum of fusariumic acid I (**1**).

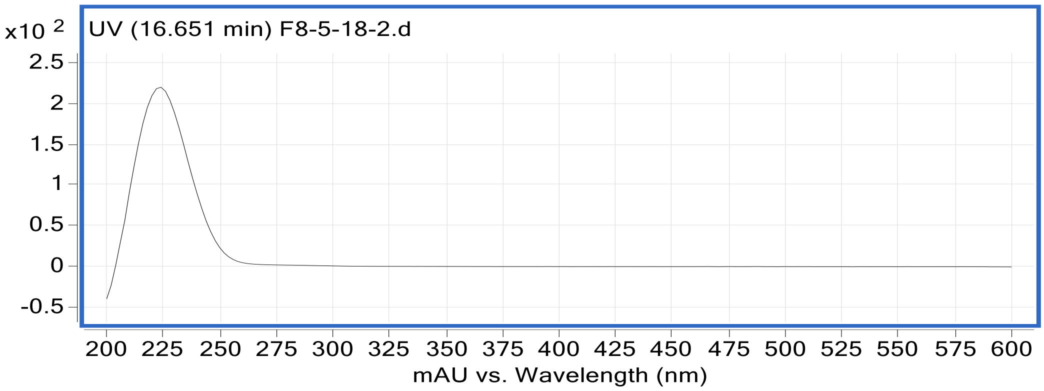

Figure S2. The UV spectrum of fusariumic acid I (**1**).

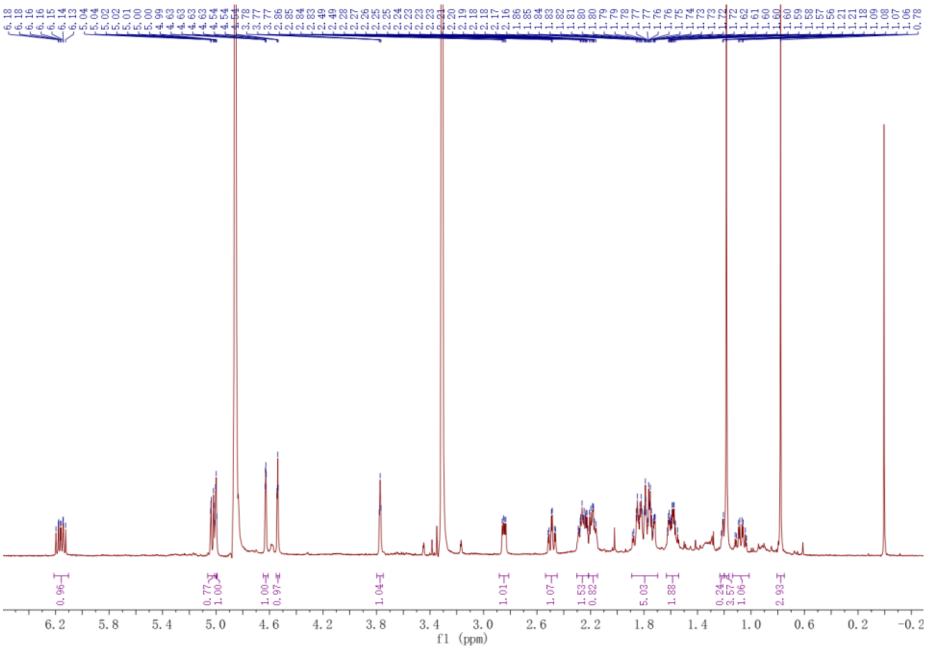

Figure S3. The ^{1}H NMR spectrum of fusariumic acid I (**1**).

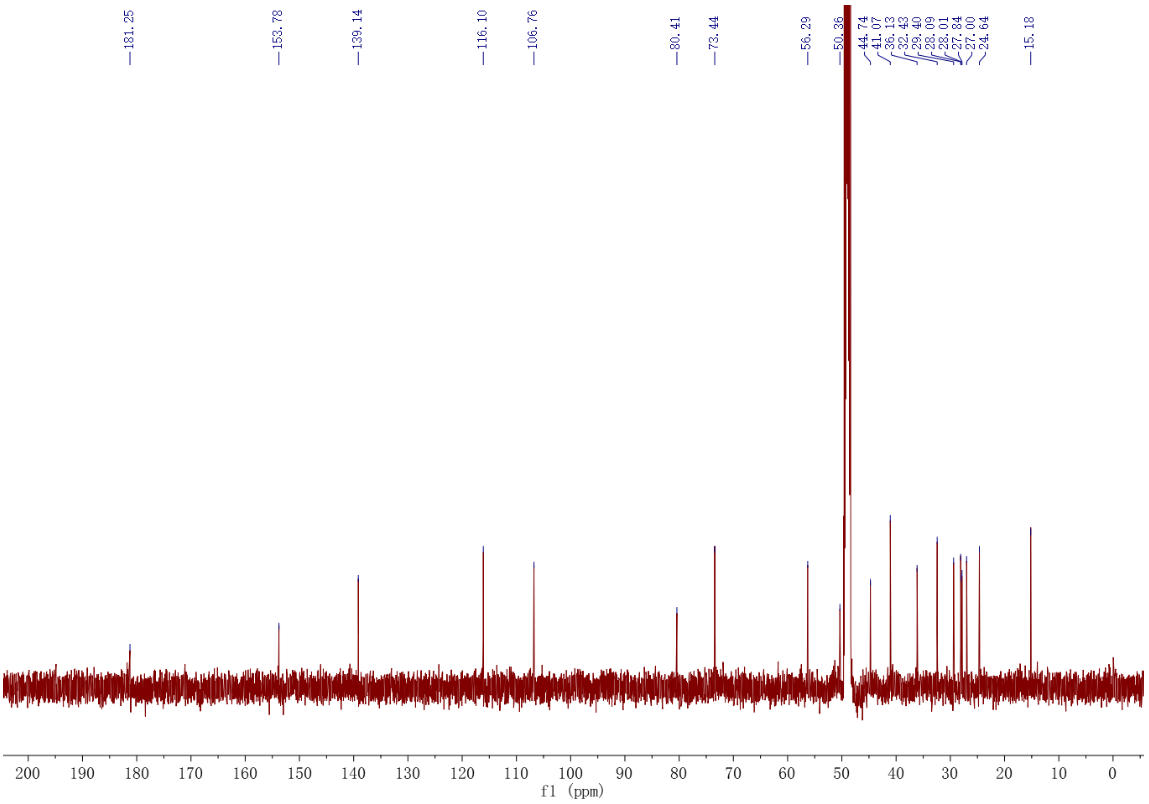

Figure S4. The ^{13}C NMR spectrum of fusariumic acid I (**1**).

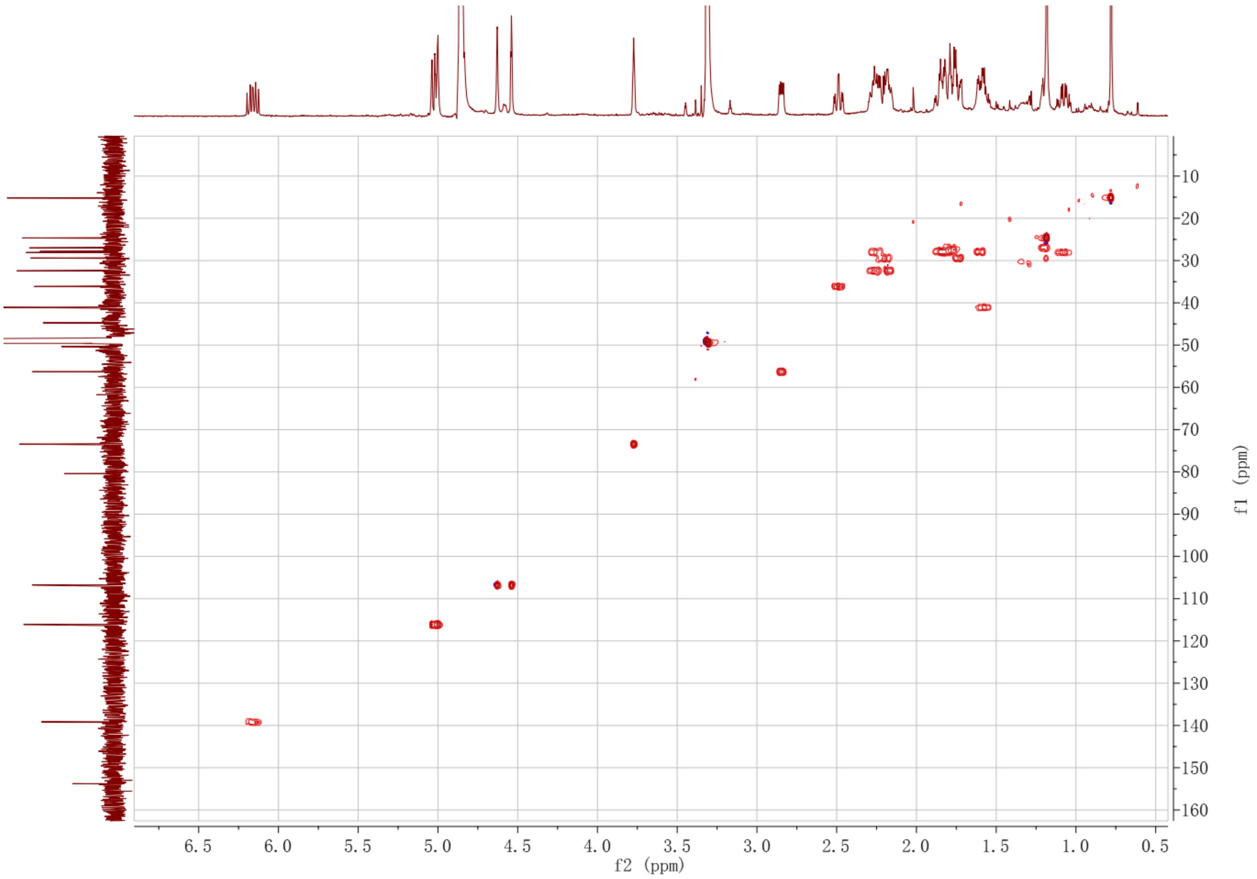

Figure S5. The HSQC spectrum of fusariumic acid I (**1**).

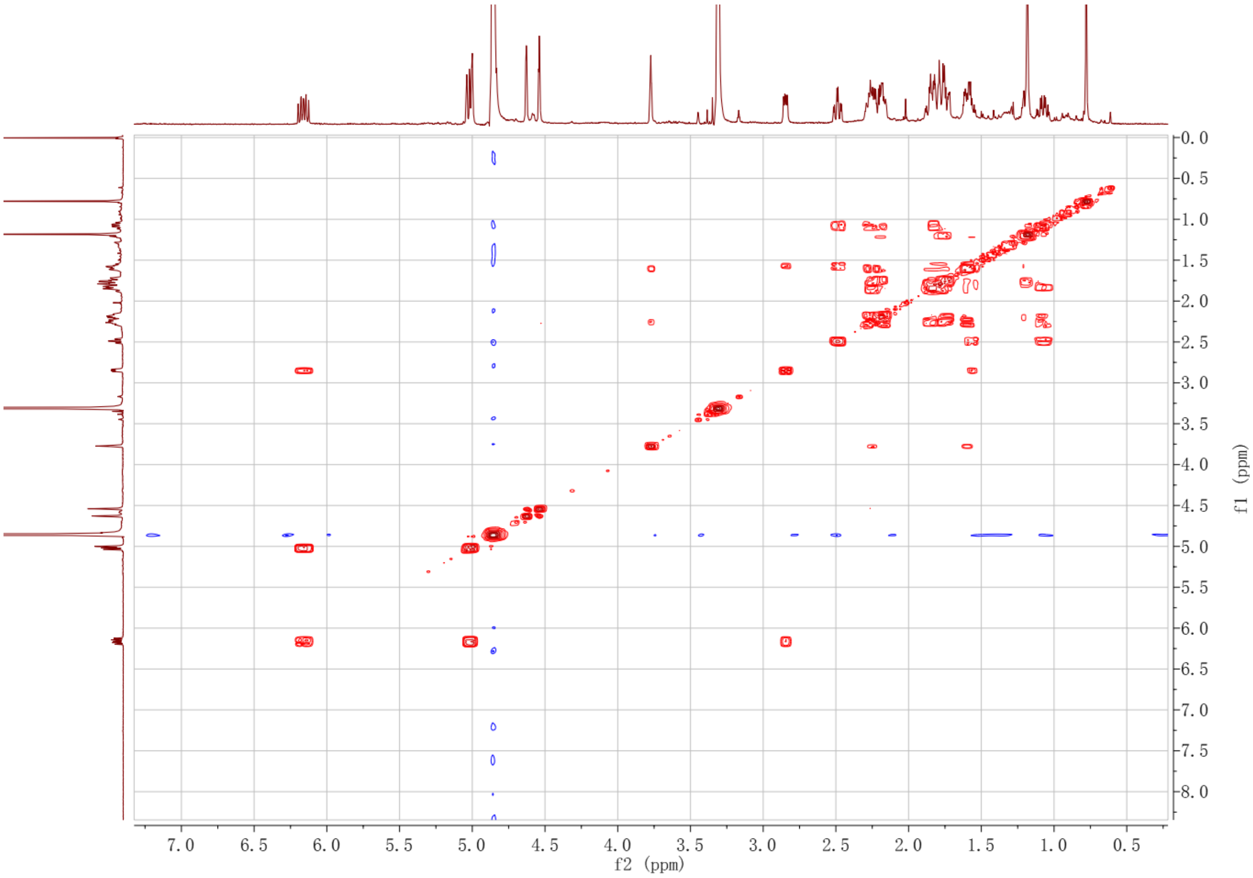

Figure S6. The ^{1}H–^{1}H COSY spectrum of fusariumic acid I (**1**).

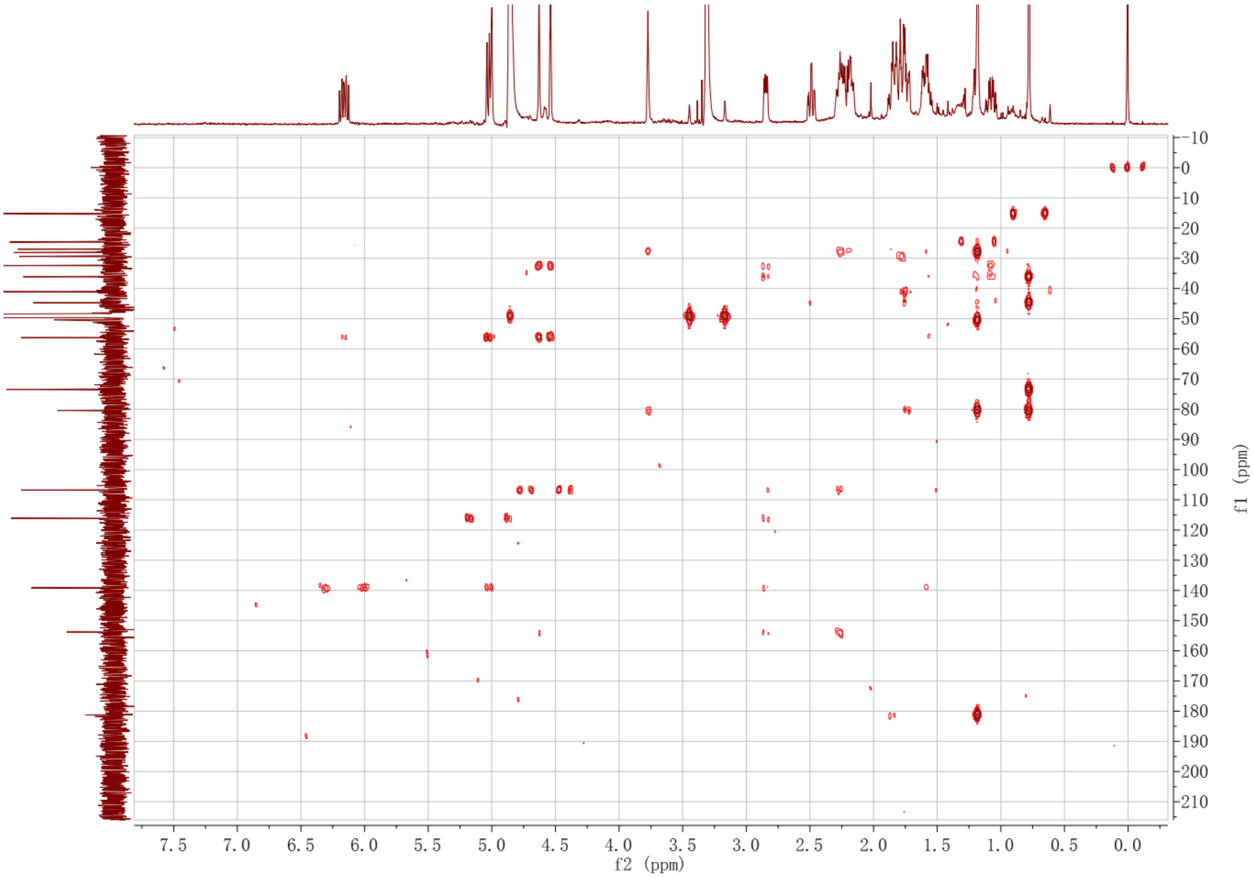

Figure S7. The HMBC spectrum of fusariumic acid I (**1**).

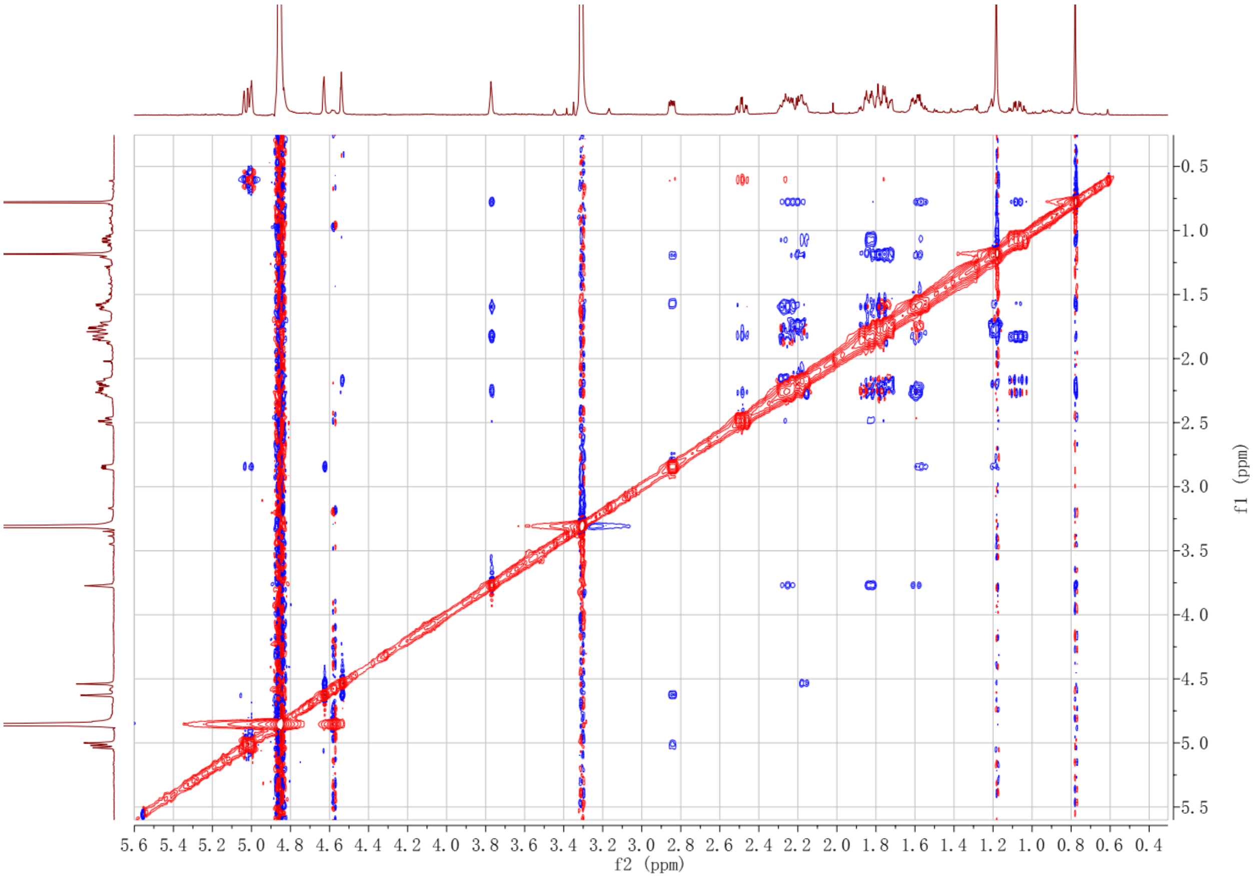

Figure S8. The NOESY spectrum of fusariumic acid I (**1**).

2. The HRESIMS, UV, 1D and 2D NMR spectra of fusariumic acid J (2)

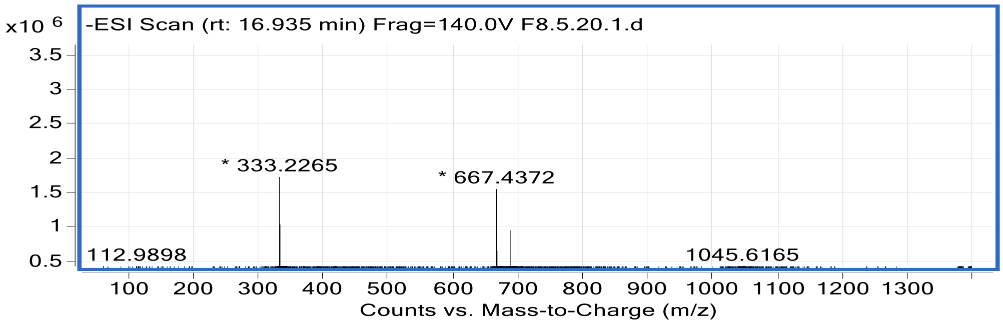

Figure S9. The HRESIMS spectrum of fusariumic acid J (**2**).

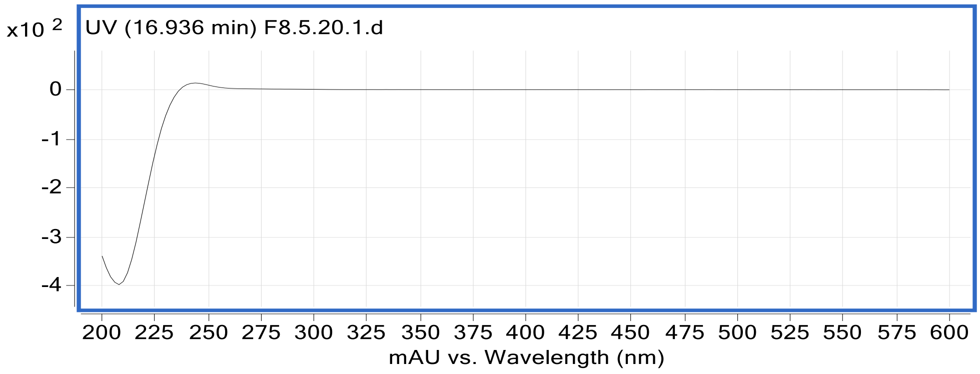

Figure S10. The UV spectrum of fusariumic acid J (**2**).

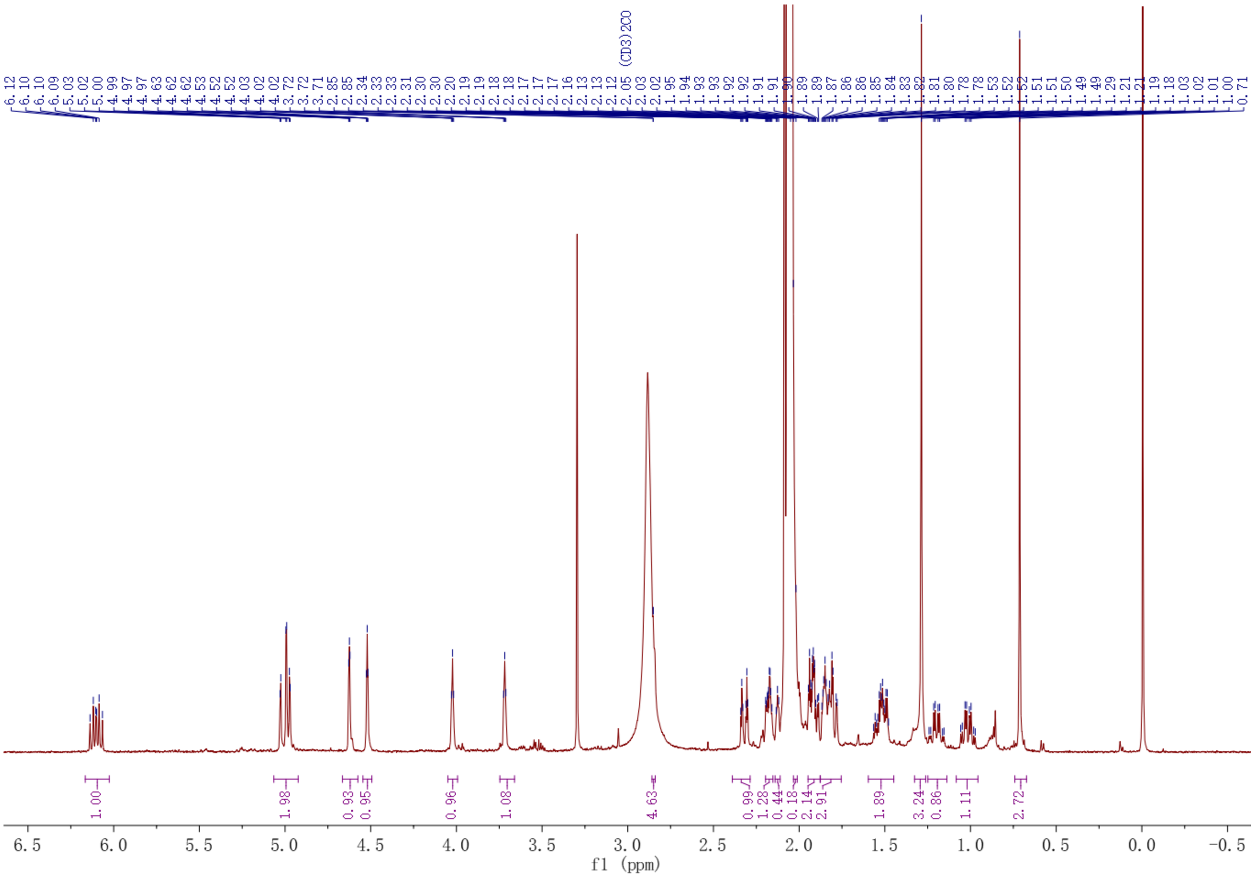

Figure S11. The ^{1}H NMR spectrum of fusariumic acid J (**2**).

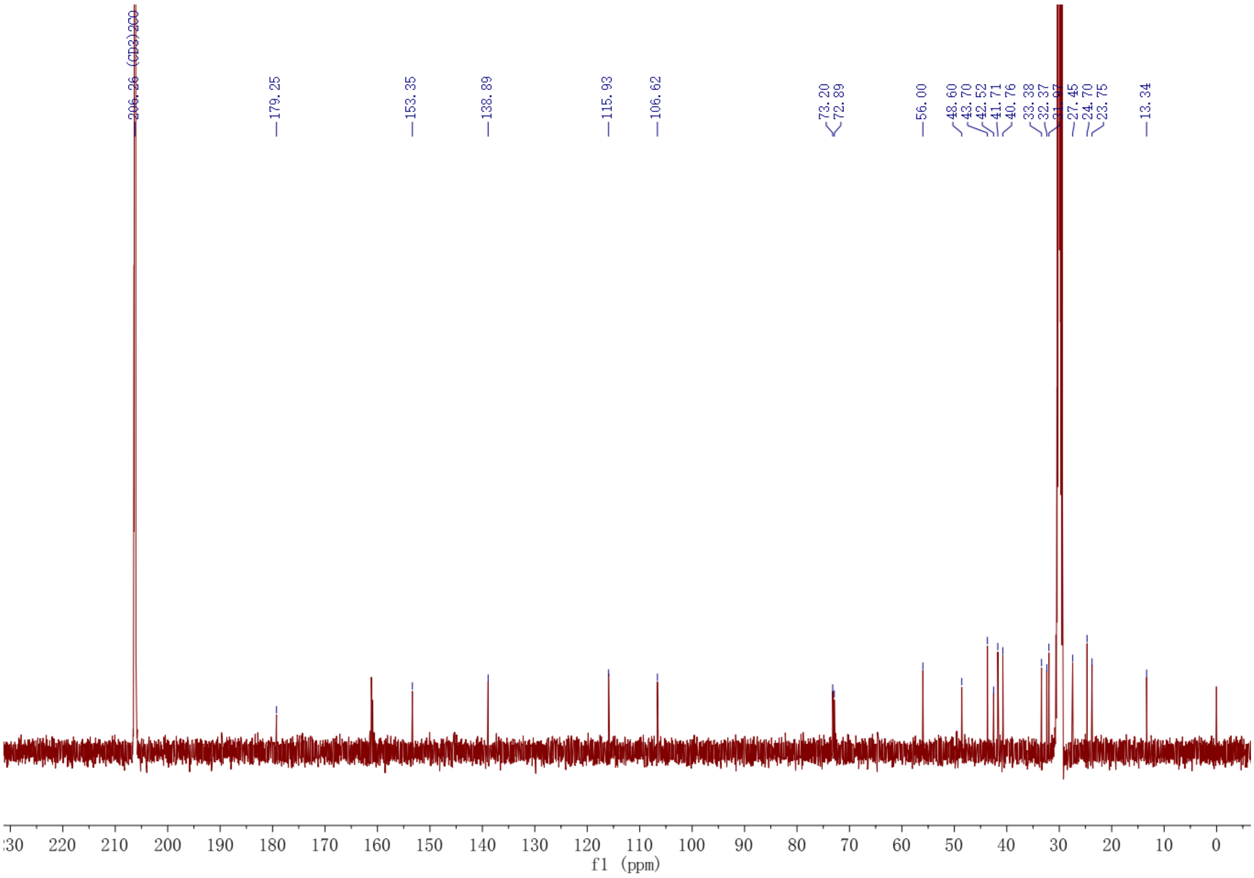

Figure S12. The ^{13}C NMR spectrum of fusariumic acid J (**2**).

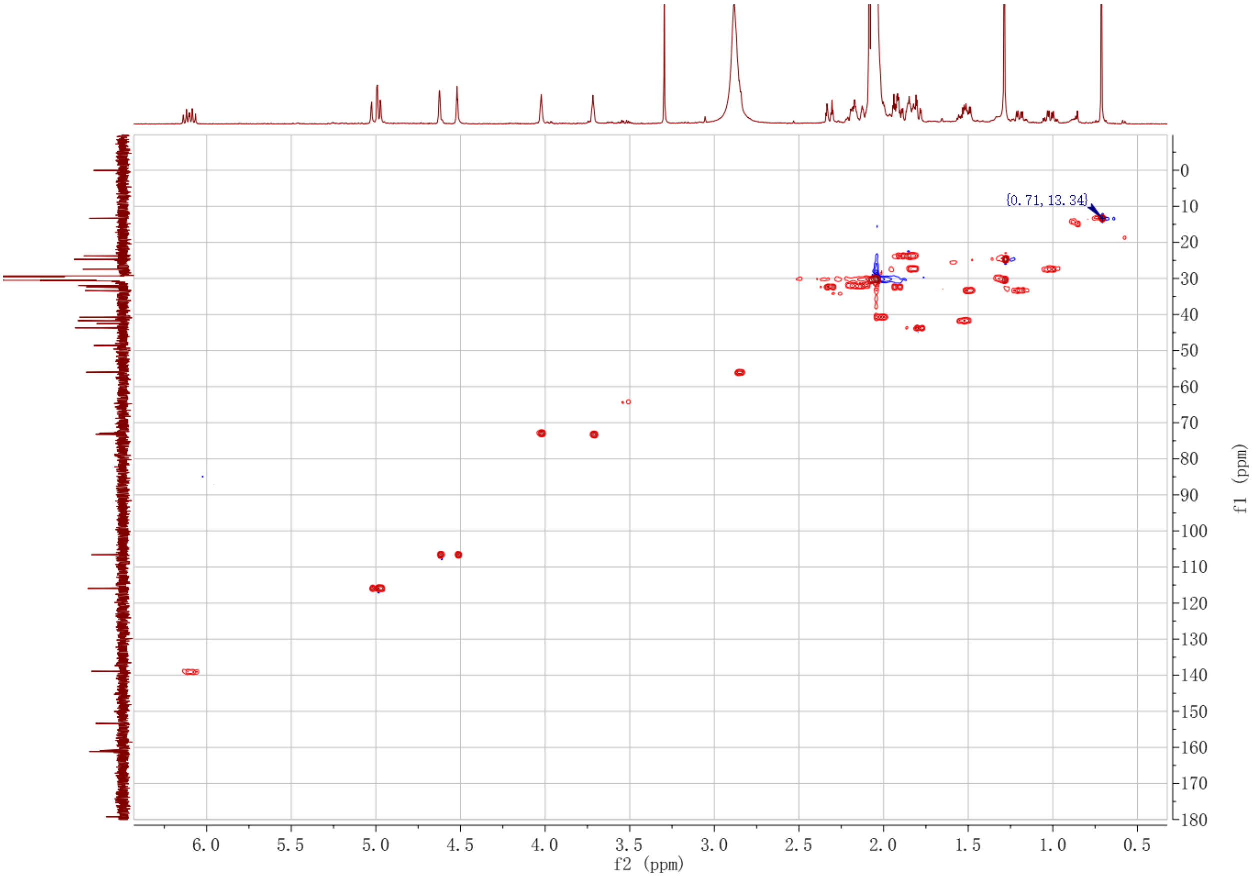

Figure S13. The HSQC spectrum of fusariumic acid J (**2**).

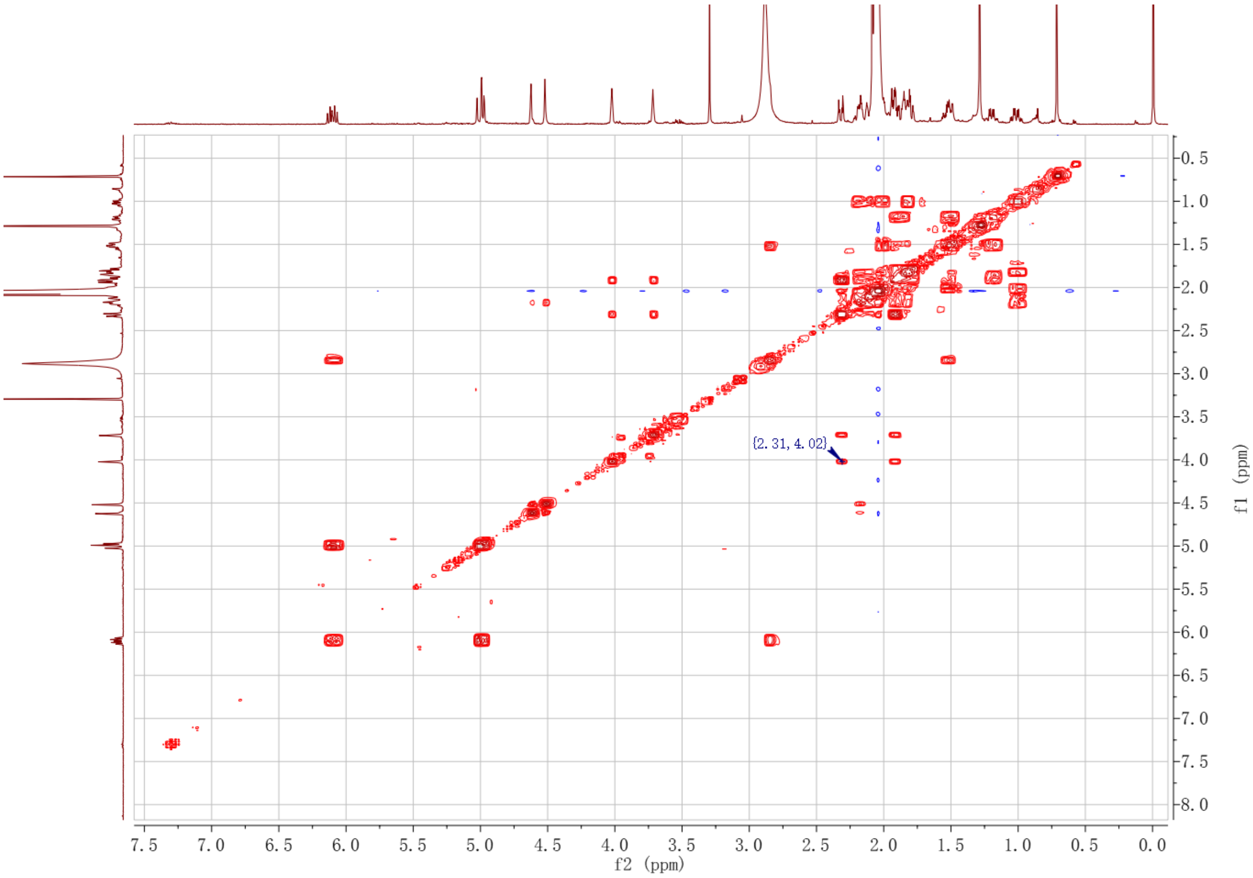

Figure S14. The ^{1}H–^{1}H COSY spectrum of fusariumic acid J (**2**).

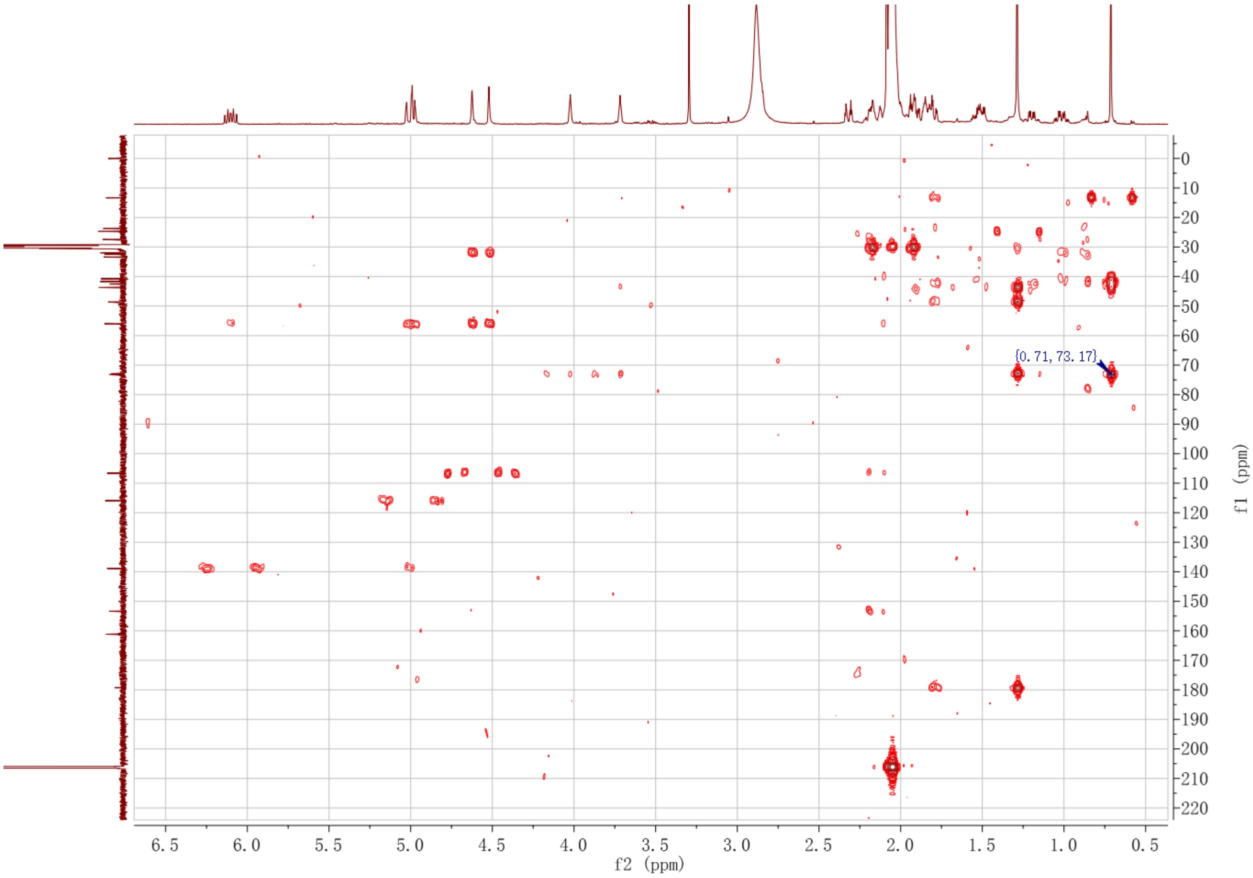

Figure S15. The HMBC spectrum of fusariumic acid J (**2**).

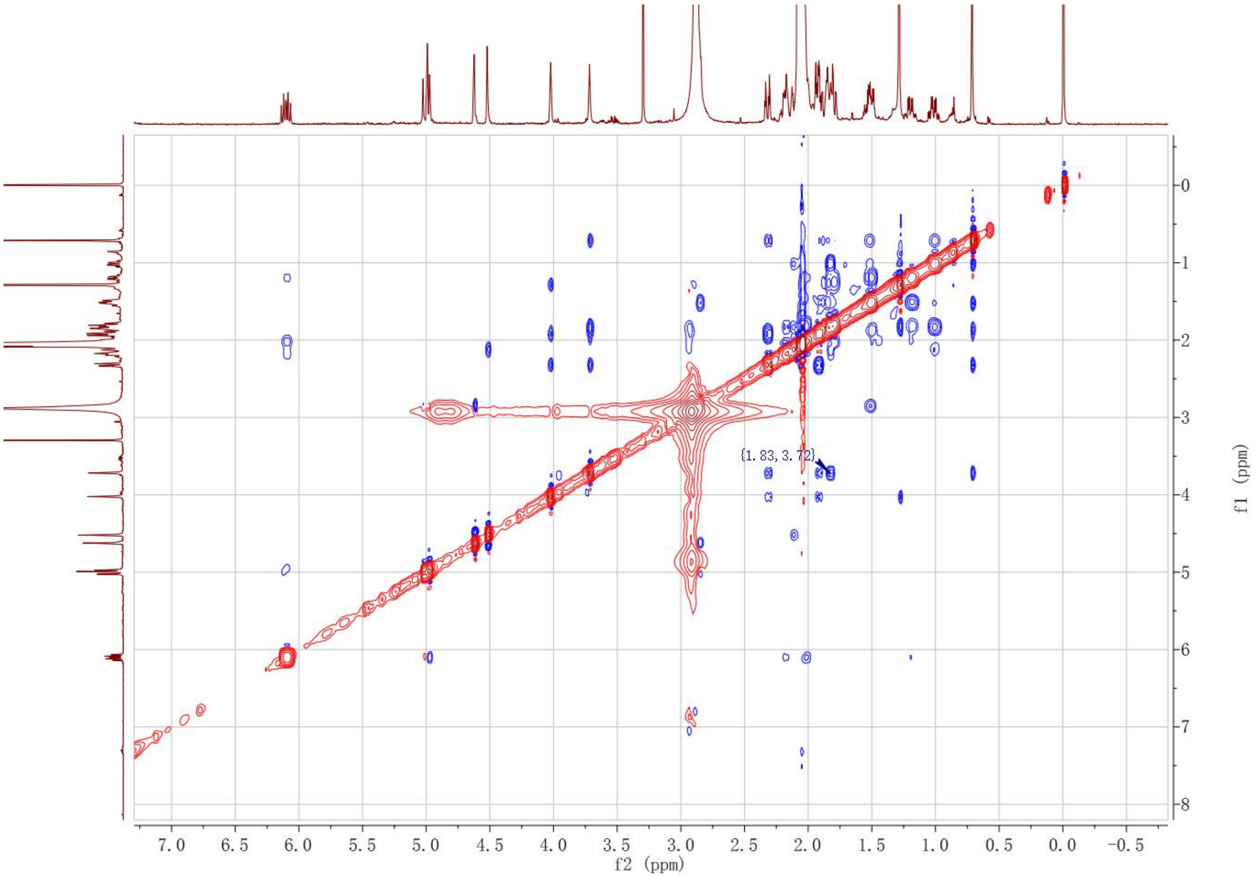

Figure S16. The NOESY spectrum of fusariumic acid J (**2**).

3. The phytotoxic and cytotoxic activities of fusariumic acids I (1) and J (2)

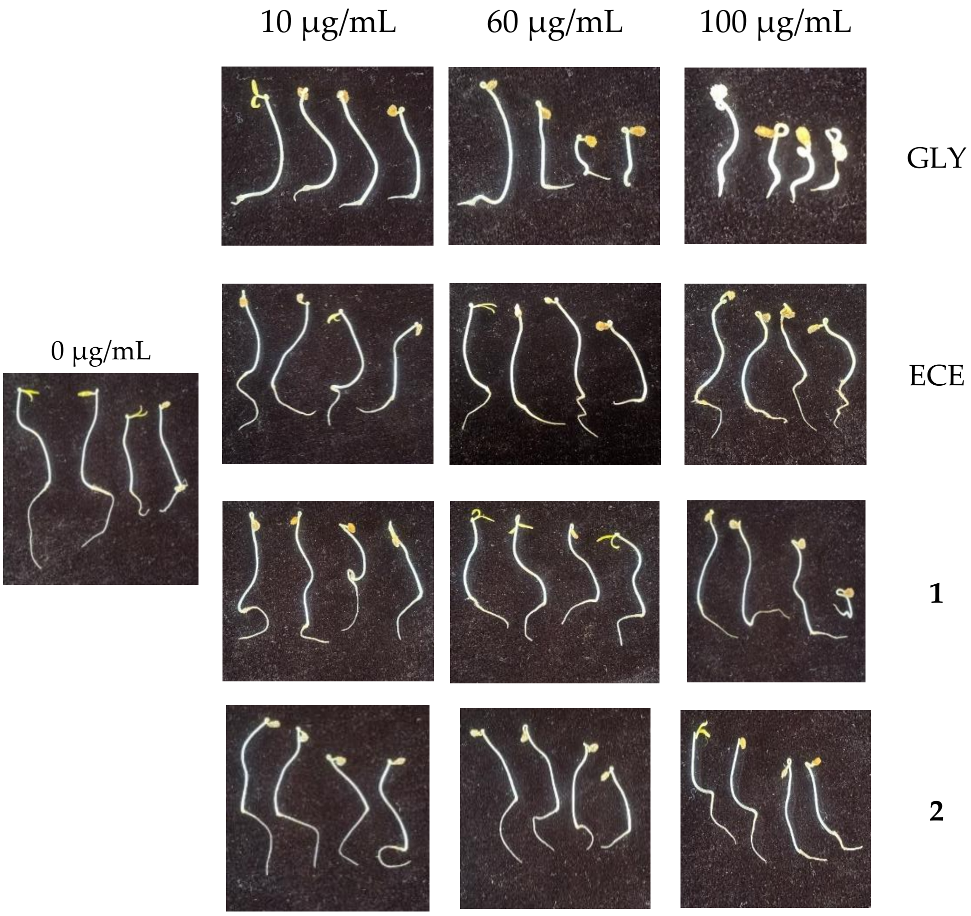

Figure S17. The effects of fusariumic acids I (**1**) and J (**2**), EtOAc crude extract (ECE) and glyphosate (GLY) on the growth of tomato seedlings for a period of 5 days.

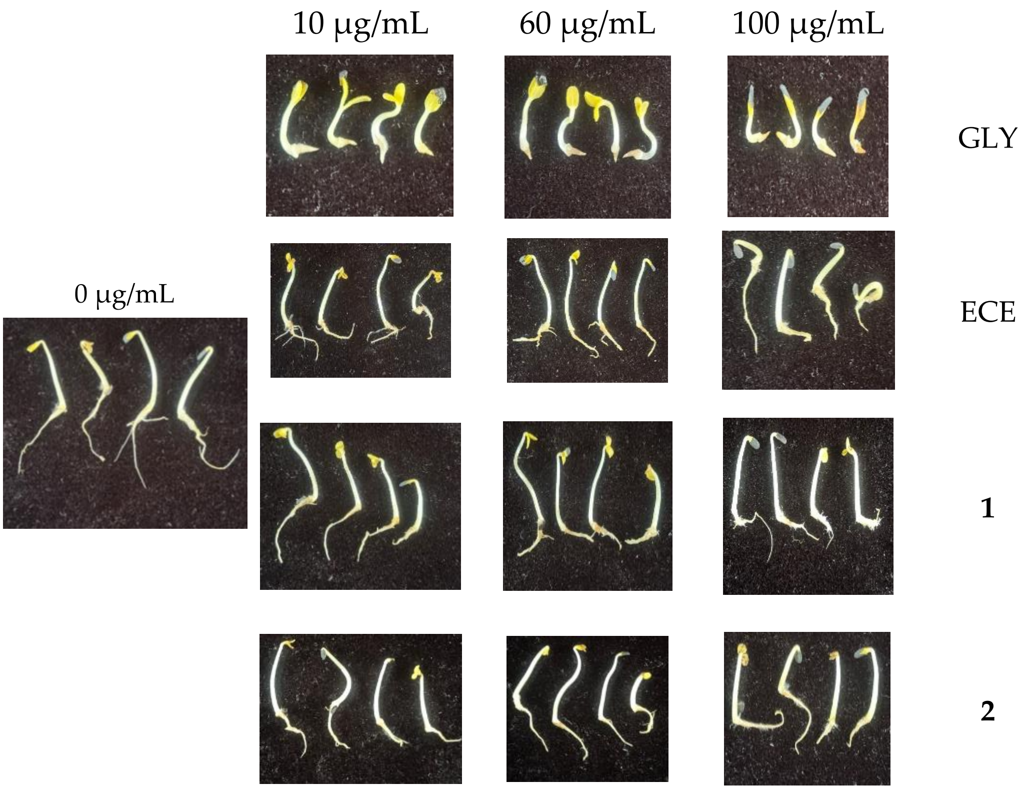

Figure S18. The effects of fusariumic acids I (**1**) and J (**2**), EtOAc crude extract (ECE) and glyphosate (GLY) on the growth of sesame seedlings for a period of 6 days.

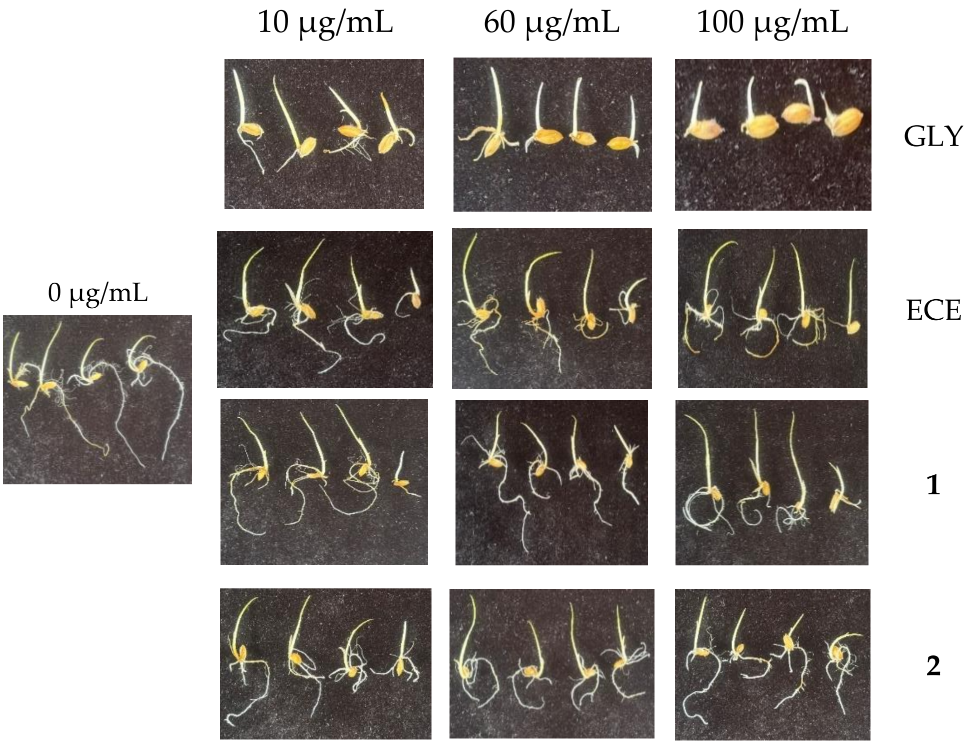

Figure S19. The effects of fusariumic acids I (**1**) and J (**2**), EtOAc crude extract (ECE) and glyphosate (GLY) on the growth of rice seedlings for 7 days.

Table S1. The cytotoxic activities of fusariumic acids I (**1**) and J (**2**) on human cancer cell lines.

**Compound**	**IC_{50} (μmol/L)**					
	**HCT116**	**U87 MG**	**BGC823**	**HepG2**	**PC-9**	**PANC1**
Fusarium acid I (**1**)	>50.0	>50.0	>50.0	>50.0	>50.0	>50.0
Fusarium acid J (**2**)	>50.0	>50.0	>50.0	>50.0	>50.0	>50.0
Taxol	0.000205	0.0281	0.00395	0.000185	0.0000701	0.00360

Note: Taxol was used as the positive control. Human cancer cell lines included colon carcinoma (HCT116), glioblastoma (U87 MG), gastric carcinoma (BGC823), hepatocellular carcinoma (HepG2), lung adeno-carcinoma (PC-9), and pancreatic carcinoma (PANC1) cell lines.
